# Forming nanoparticles of water-soluble ionic molecules and embedding them into polymer and glass substrates

**DOI:** 10.3762/bjnano.3.30

**Published:** 2012-03-21

**Authors:** Stella Kiel, Olga Grinberg, Nina Perkas, Jerome Charmet, Herbert Kepner, Aharon Gedanken

**Affiliations:** 1 Department of Chemistry, Kanbar Laboratory for Nanomaterials, Nanotechnology Research Center, Institute of Nanotechnology and Advanced Materials, Bar-Ilan University, Ramat-Gan, 52900, Israel; 2 HES-SO Arc, Institut des Microtechnologies Appliquées, Eplatures-Grises, 1 7, 2300 La Chaux-de Fonds, Switzerland

**Keywords:** deposition, ionic salt nanoparticles, parylene, sonochemistry

## Abstract

This work describes a general method for the preparation of salt nanoparticles (NPs) made from an aqueous solution of ionic compounds (NaCl, CuSO_4_ and KI). These nanoparticles were created by the application of ultrasonic waves to the aqueous solutions of these salts. When the sonication was carried out in the presence of a glass microscope slide, a parylene-coated glass slide, or a silicon wafer the ionic NPs were embedded in these substrates by a one-step, ultrasound-assisted procedure. Optimization of the coating process resulted in homogeneous distributions of nanocrystals, 30 nm in size, on the surfaces of the substrates. The morphology and structure of each of the coatings were characterized by physical and chemical methods, such as X-ray diffraction (XRD), scanning electron microscopy (SEM), atomic force microscopy (AFM), Raman spectroscopy and X-ray photoelectron spectroscopy (XPS). After 24 h of leaching into water the nanoparticles of the inorganic salts were still present on the slides, and complete leaching of nanoparticles occurred only after 96 h. A mechanism of the ultrasound-assisted coating is proposed.

## Introduction

The incorporation of nanocrystals into dielectric matrices, such as glass or polymers, has become a topic of broad interest in recent years. The research in the area of nanostructured composites is aimed at studying their fundamental properties as well as applications in tissue engineering, nanooptics and nanoelectronics [1–5].

Unlike the synthesis of NPs of metal oxides, metal chalcogenides, and even some metal fluorides, the synthesis of NPs of water-soluble ionic compounds has not reached the same level of attention, and only a very few publications are found [6]. This current paper describes a general method for the preparation of water-soluble ionic NPs. To help prove the formation of these NPs they were examined both in the solution in which they were formed as well as in solid matrices. They were embedded in the solid substrate by the sonochemical method, which was performed subsequently to their formation.

Various methods have been used for the incorporation and growth of arrays of nanocrystals on or embedded into substrate hosts. One of these methods is chemical bonding. For example, the cross-linking process was used for the embedding of NaCl nanocrystals into polymers containing unsaturated double bonds [1–2].

Some other methods were developed for the coating of polymers with nanocrystals of NaCl, Na_2_CO_3_ and Na_2_SO_4_, exploiting the difference in wettability between the regions of the patterned polymeric substrate [3]. Suh et al. [3] fabricated single nanocrystal arrays of various sizes on sub-microwells of poly (ethylene glycol) copolymer, using selective wetting of the hydrophilic regions of the exposed substrate surface and subsequent drying. The single NaCl nanocrystals in the range of 100–150 nm were prepared by immersion of a concave-patterned polystyrene film in aqueous NaCl solution, which was then lifted out of the solution slowly [4]. A new synthesis route was developed by Malfatti et al. for the formation of NaCl nanoboxes on a silicon wafer and silica glass slide as substrate, through an evaporation-induced self-assembly process [7].

The insertion of nanocrystals into polymer and glass substrates was also performed by some physical methods. The incorporation of NaCl nanocrystals into porous glass by high-pressure injection was reported by Parfen’eva et al. [5], and a change of the thermal conductivity of the substrate was demonstrated. The embedding of NaCl nanoparticles into a polymeric film was recently realized by the use of an electromagnetic field [8]. According to this method, the nanoparticles absorbed energy upon illumination by laser light, converted it to heat, and sank into a locally softened polymer film, as probed by atomic force microscopy. Lomonosov et al. [9] used an ultrasonic piezo dispenser for the introduction of NaCl nanoparticles from 1 M aqueous NaCl onto mica slides and demonstrated regions with a regular NaCl nanocrystal structure, by AFM. These structures varied from rows of single square nanocrystals to rows of long rectangular crystals with average sizes of 300 nm in width, 60 nm in height and up to several micrometers long. The structural changes were explained as being due to surface diffusion; however, the mechanism of the deposition, the attachment of NaCl nanoparticles and the formation of the crystalline structure were not explicitly discussed [9].

We have reported on the sonochemical deposition of inorganic nanoparticles on and into various materials and demonstrated that the ultrasound assisted method is effective in uniform distribution and strong attachment of nanoparticles to the surfaces of the substrates and even their penetration into the solid body [10]. These observations can be explained as a result of the extreme conditions of high pressure (>1000 atm) and temperature (>500 K) developed during the collapse of the acoustic bubble. The after effect of this collapse near a solid surface is the formation of high-speed microjets and shock waves in the liquid, directed towards the solid surface. These microjets throw the just-formed nanoparticles toward the substrate at such a high speed (>500 m/s) that it leads to the strong adherence of the NPs to the surface without the need for any binding agents [11–12].

In the present work we report for the first time on a novel general method for the preparation of water-soluble NPs of ionic salts. The method was applied to NaCl, CuSO_4_, and KI. The general method suggested for the preparation of water-soluble ionic NPs is sonochemistry. The salt nanocrystals of NaCl, CuSO_4_ and KI were immobilized from the aqueous solution of the dissolved ionic compounds onto the microscope glass slides, parylene-coated glass slides, and silicon wafers. This was accomplished by a one-step, ultrasound-assisted procedure. The ionic nanoparticles were thrown to the solid surface by the sonochemical microjets and were strongly anchored to the substrates. The coated substrates were characterized by chemical and physical methods.

## Experimental

### Materials

All of the chemical reagents were purchased from Sigma Aldrich and were of analytical chemical purity and used without further purification. The glass microscope slides were purchased from Laborderate GmbH Germany. The parylene-C-coated glass microscope slides were received from COMELEC SA (Switzerland), and the procedure for their preparation has been described previously [13]. The thickness of the parylene layer was in the range of 6–12 μm. Each glass slide was cut to a size of 1 inch^2^ to be inserted into the reaction vessel.

#### Deposition of NaCl/CuSO_4_/KI on regular/parylene-coated glasses and on silicon wafer/parylene-coated silicon wafer

**Figure 1:**
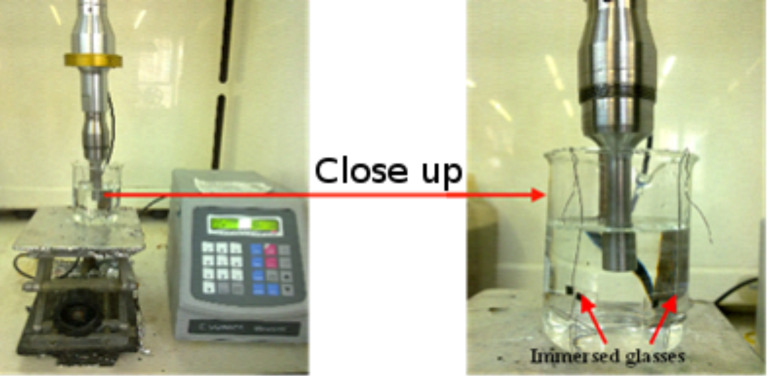
Coating installation.

Typically, NaCl was dissolved in doubly distilled water to obtain a concentration in the range of 0.04–0.5 M. The dissolution of the NaCl, CuSO_4_ and KI was achieved by magnetic stirring of the solutions for 30 min at room temperature. The sonochemical treatment was carried out in the presence of glass/parylene-coated glass by immersion of the high-intensity Ti horn (20 kHz, 750 W) into the reaction vessel (Figure 1). The reaction time varied from 15 min to 90 min. The efficiency of the ultrasonication was in the range of 23–30%. The deposition of CuSO_4_ and KI was achieved by the same procedure. All the experiments were repeated three times and good reproducibility of the results was demonstrated.

#### Characterization

The concentrations of ions in the sonicated solutions were determined by volumetric titration using Mohrs [14] and complexometric EDTA reagent [15].

The structure of the deposited nanoparticles was characterized by XRD on a Bruker D8 diffractometer (with Cu Kα = 1.5418 Å radiation). The size of the particles in the working solution was controlled by DLS measurements using a Coulter particle analyzer (Malvern Zetasizer). The morphology of the glasses coated with NaCl/CuSO4/KI nanoparticles was studied by SEM using the JEOL-JSN 7000F device. The AFM measurements and imaging were carried out with a Nanoscope V Multimode scanning probe microscope (Digital Instruments, Santa Barbara, CA). All the images were obtained in contact mode with a single NP silicon nitride probe (force constant of 0.58 N/m, Digital Instruments, Santa Barbara, CA). The scan angle was maintained at 90° and the images were captured in the retrace direction at a scan rate of 1 Hz. The XPS measurements were performed on a Kratos Axis-HS spectrometer (residual gas pressure of ~5 × 10^−10^ Torr) with monochromatized Al Kα radiation (*h*υ = 1486.68 eV) and a hemispherical analyzer.

#### Leaching studies

The leaching studies were performed by placing the coated glasses in 20 mL of doubly distilled water at room temperature for different periods of time (from 2 to 96 h) under continuous stirring. The leaching of salt nanoparticles was estimated by inductively coupled plasma (ICP) analysis on the ULTIMA JY2501 device and examined with AFM.

## Results and Discussion

The current work describes the deposition of NaCl, CuSO_4_ and KI nanoparticles on different types of substrates by a one-step, ultrasound-assisted procedure. The results presented here compare the experimental data collected for the three salts. It is important to note that the applied technique is general and the obtained results appeared to be similar to all studied inorganic salts.

### Surface properties and morphology

**Figure 2:**
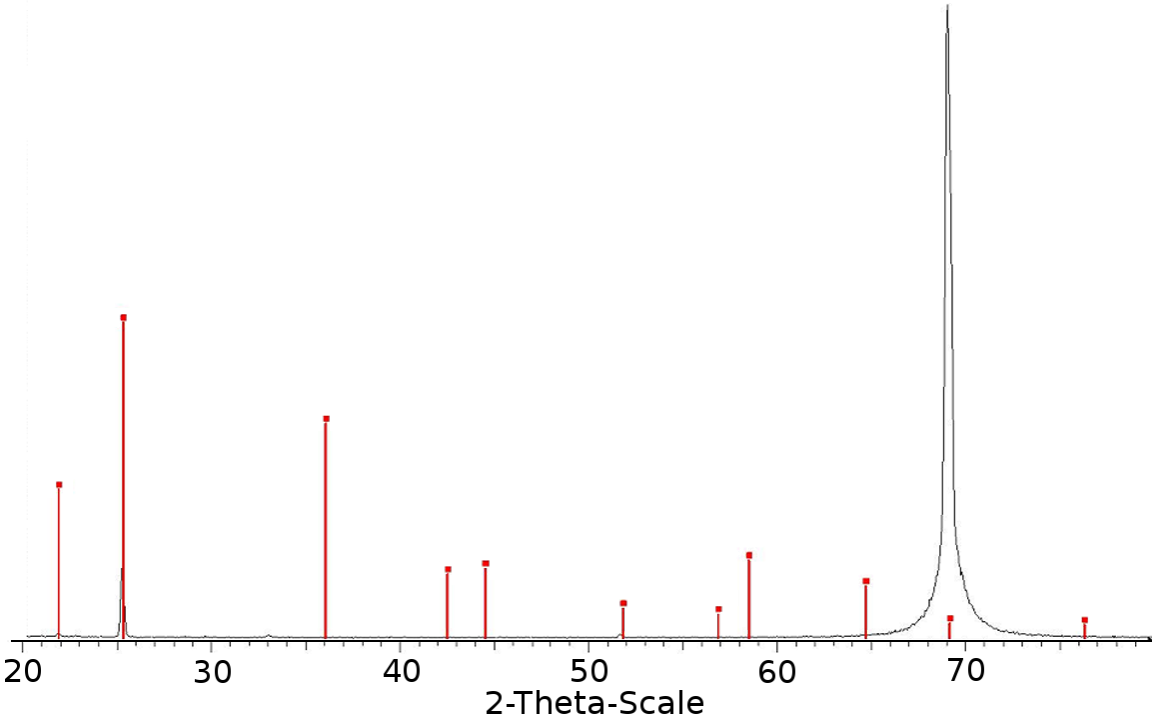
XRD diffraction pattern of NPs of KI coated on a silicon wafer (the red lines indicate literature data (PDF 00-004-0471); the black diffraction lines match well with the KI literature values).

The first aim of this research was to prove that the nanoparticles produced and deposited onto the polymer and glass substrates have the same composition as the initial salts dissolved in water before the sonication. The structural characterization of the KI nanoparticles deposited on the silicon-wafer slide was performed by XRD, and the result is presented in Figure 2.

The high-intensity peak at 69.2° is assigned to reflection lines of the silicon wafer. The peak at 25.106° corresponds to the (100) reflection line of KI (PDF 00-004-0471). However, not all of the expect peaks for crystalline KI appeared in the spectrum. The reason for not observing more than two diffraction peaks of KI may be the small quantity of deposited salt particles on the substrate (less than 4%). However, we can also speculate that a peak at 36.1°, which would be the second largest according to PDF 00-004-0471, is missing due to a directional growth of the KI on the silicon surface.

**Figure 3:**
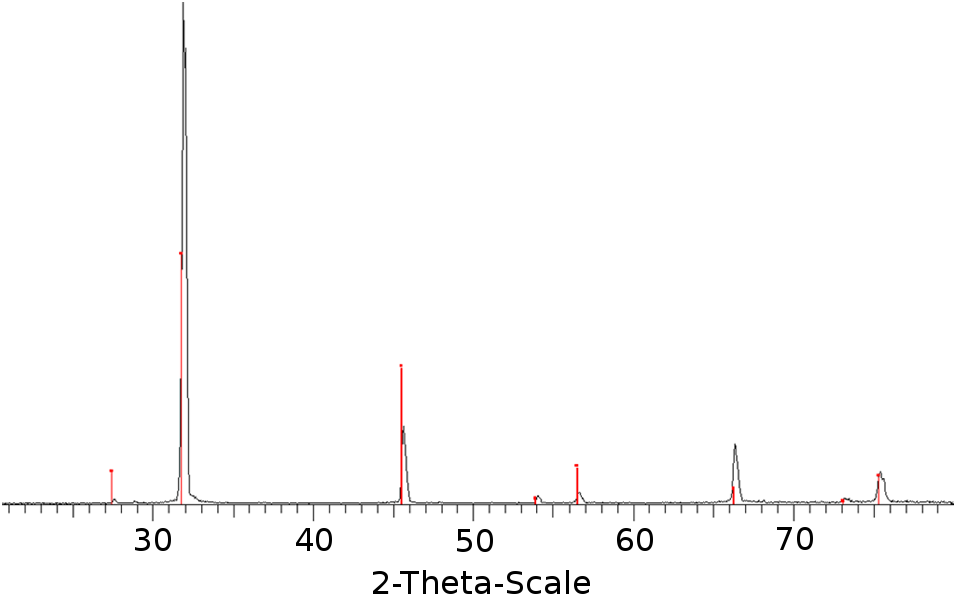
XRD diffraction pattern of NaCl precipitate collected at the end of sonication (red lines indicate the literature data of PDF 00-005-0628, the black diffraction lines were measured for the collected powder).

An additional proof of nanoparticle formation in the sonochemical reaction, based on sonication of the saturated solution of NaCl (CuSO_4_, KI), was the formation of sediment that was not immobilized on the glass slide but instead precipitated to the bottom of the reaction vessel and collected at the end of the reaction. The XRD analysis of the collected powder matched well to the diffraction peaks of the crystalline NaCl (CuSO_4_, KI). Figure 3 displays an example of XRD patterns of precipitated NaCl particles obtained at the end of the sonochemical treatment. The peaks at 2θ *=* 27.4, 31.8, 45.4, 53.8, 56.4, 66.2, 73.3 and 75.3° are assigned to the (111), (200), (220), (311), (222), (400), (331) and (420) (PDF 00-005-0628) reflection lines corresponding to the face-centered-cubic structure of NaCl. No peaks characteristic of any impurities were detected. The intensity of the 31.8, 45.4, 53.8, and 56.4° diffraction peaks follow the literature values indicated by the red lines. The diffraction peaks at 66.2, and 75.3° deviate from the literature values. This may be a result of (a) another polymorph with (nearly) the same lattice spacings but different electron distribution, or (b), more likely, the possibility that the positional correlation length is different for different lattice directions. In other words, a directed growth of NaCl crystals emerging as, for example, a fibrillar structure may explain these stronger-than-expected intensities.

**Figure 4:**
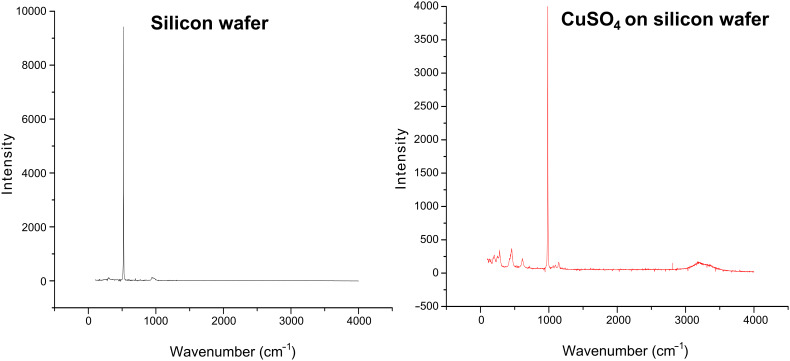
Raman spectrum of a silicon wafer and a silicon wafer coated with CuSO_4_.

The Raman measurements were performed in order to prove the presence of salts on the glass substrates. Figure 4 demonstrates the Raman spectra of the silicon wafer coated with CuSO_4_. The lines between 200 and 450 cm^−1^ are assigned to the internal modes of the complex ions CuSO_4_. The peaks at 475 cm^−1^, 612 cm^−1^ and 1107 cm^−1^ are assigned to the vibration frequencies of (SO_4_)^−2^. In the unit cell of CuSO_4_, the water molecules occupy three different sites and their geometry is nearly that of the free water molecule (3210 cm^−1^) [16].

**Figure 5:**
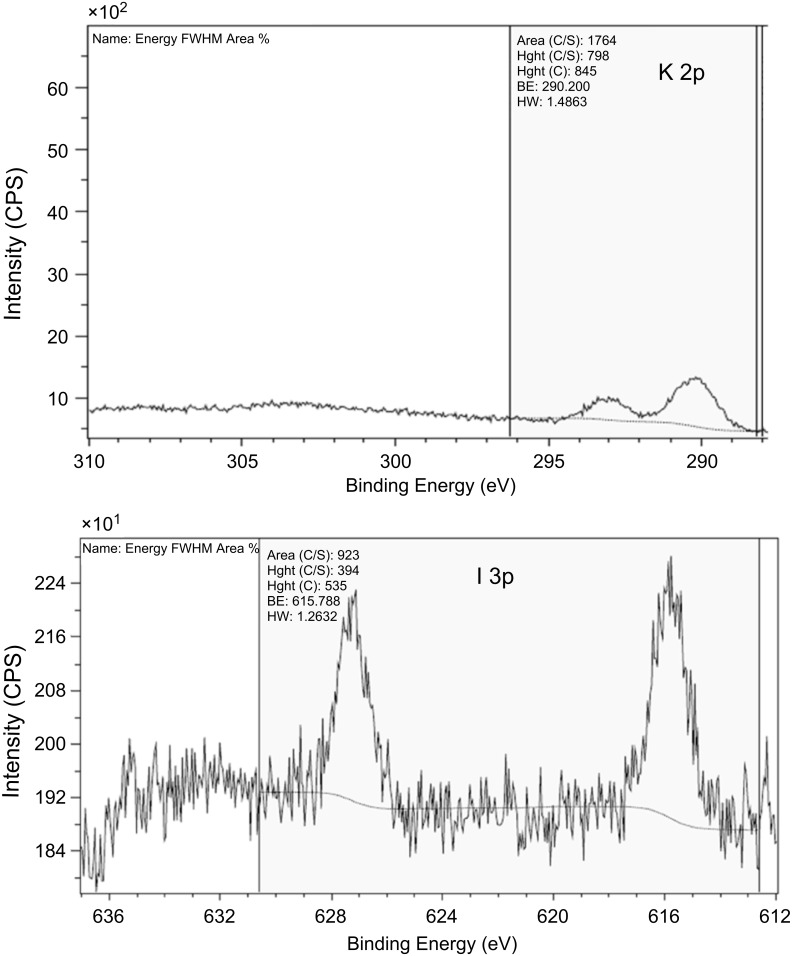
K (top) and I (bottom) XPS spectra detected from the glass coated with KI.

We also performed the XPS studies of the coated and uncoated glass slides, and the results are presented in Figure 5. There was no use in implementing XPS on the slides coated with NaCl, because the glass itself also contained both Na and Cl atoms. Figure 5 demonstrates the XPS spectrum of glass coated with KI nanoparticles. In this case the presence of K and I can only originate from KI, and this result indicated that the salt nanoparticles were attached to the surface of the glass substrate. Similar results were obtained with the samples coated by CuSO_4_.

To check whether there are differences in the concentrations of ions in the solutions before and after sonication, volumetric titration according to Mohrs method (Cl^−^) [14] and complexometric titration with EDTA reagent (Cu^2+^) [15] were performed. The titrations were carried out for the two types of solutions: The solutions that were sonicated without the immersed glass slides and the solutions that were sonicated in the presence of the immersed glass slides. The results obtained for the solutions sonicated without the immersed slides did not show any difference in the concentrations of Cl^−^ or Cu^2+^ ions compared with their concentrations in the initial solution. At the same time, the titration of the solution sonicated with the immersed slides revealed a decrease in the concentrations of Cl^−^ (for NaCl) and Cu^2+^ ions (for CuSO_4_) in the solutions comparing to their concentrations in the initial solution. This means that part of the solute was retained on the substrate as a deposited layer.

**Figure 6:**
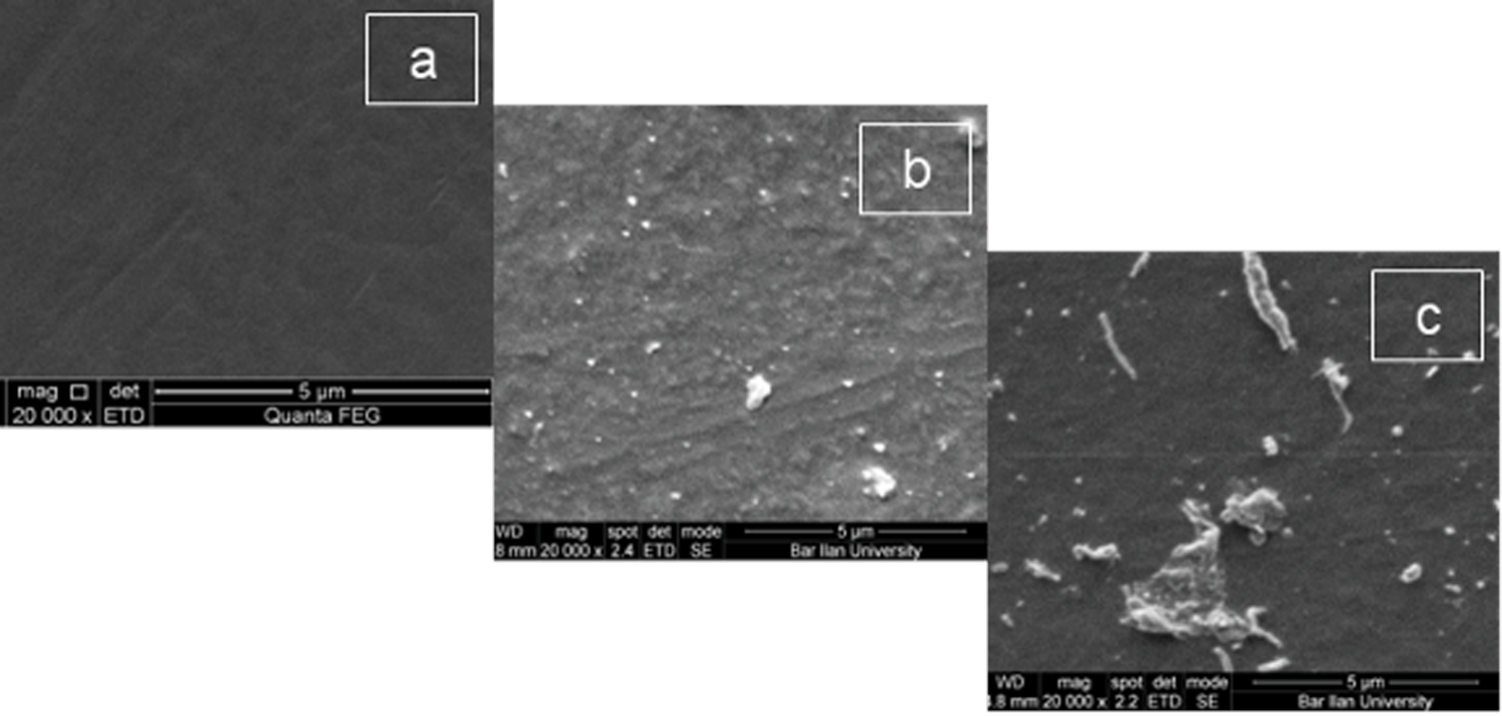
The SEM images of (a) pristine microscope glass slide (magnification 20000×); (b) the glass slide coated with NaCl nanoparticles after 30 min reaction in a mother solution of 0.08 M concentration (magnification 20000×); (c) the glass slide coated with NaCl nanoparticles after 30 min reaction in a mother solution of 0.3 M concentration (magnification 20000×).

In order to achieve a homogeneous distribution of nanoparticles on the surface, optimization of the sonochemical process was performed by varying the concentration of the reagents and the time for the sonochemical reaction. The influence of the reaction parameters on the obtained results was examined by the SEM method. When the concentration of the starting solution was increased with the other parameters kept unchanged, growth of the deposited particles was observed. For instance, when the concentration of NaCl in the initial solution was 0.08 M the average size of the deposited nanoparticles was 40 nm (Figure 6b), whereas when the concentration of the mother solution was increased to 0.3 M larger particles and agglomerates were obtained (Figure 6c).

**Figure 7:**
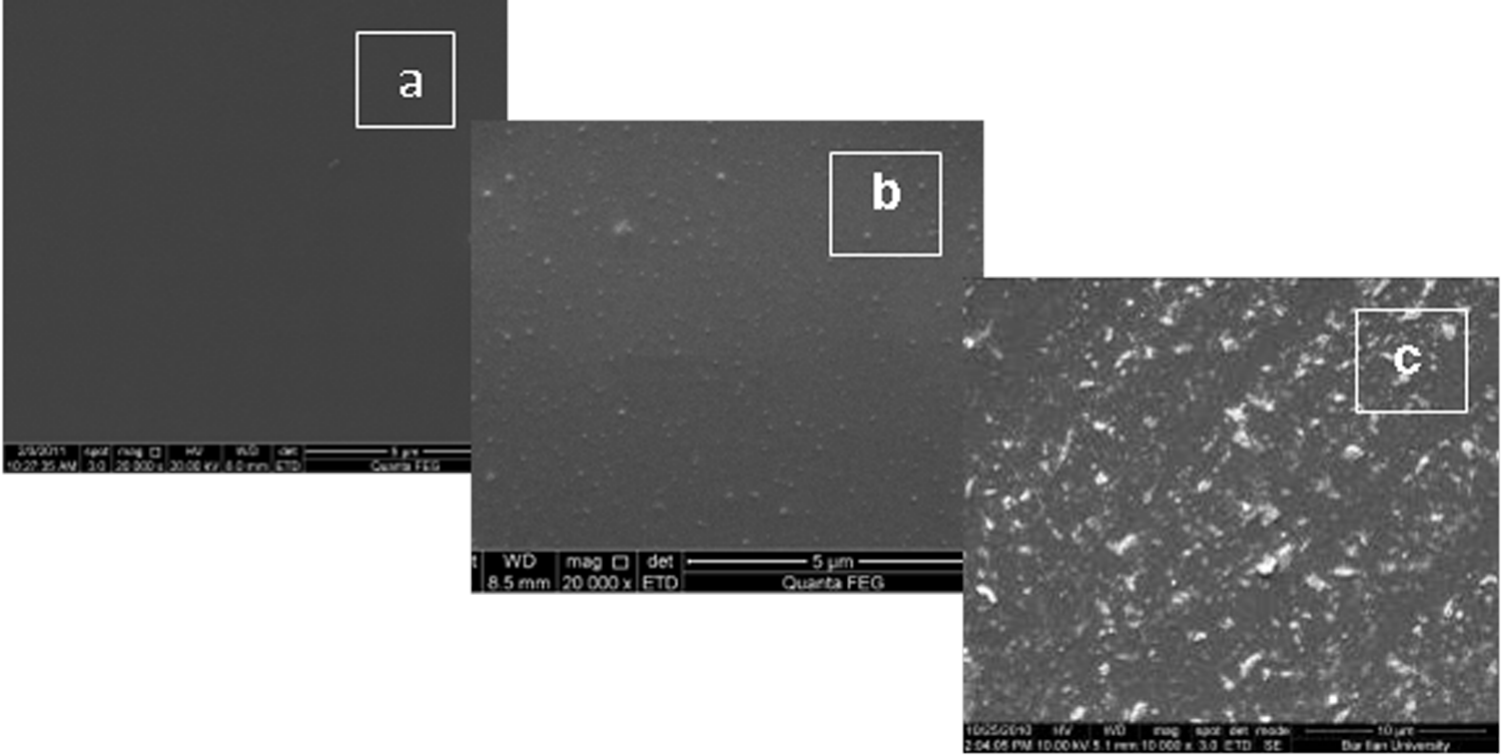
SEM images of (a) parylene-coated glass slide (magnification 20000×); (b) parylene-coated glass slide coated with CuSO_4_ nanoparticles after 30 min reaction in a mother solution of 0.125 M concentration (magnification 20000×); (c) parylene-coated glass coated with CuSO_4_ particles after 90 min reaction in a mother solution of 0.125 M concentration (magnification 10000×).

Another factor that influenced the size of the nanoparticles and the morphology of deposition was the sonication time. Images in Figure 7b and Figure 7c present CuSO_4_ particles deposited on parylene-coated glass slide from a 0.125 M mother solution, but in the case of Figure 7b the exposure time was 30 min and in Figure 7c 90 min. This obviously shows that an increase in the sonication time leads to enlarged particles and the appearance of aggregates. It was found that the most homogeneous coating could be obtained when the initial concentration of inorganic salts was in the range of 0.05–0.125 M and the ultrasound treatment lasted for 30 min.

**Figure 8:**
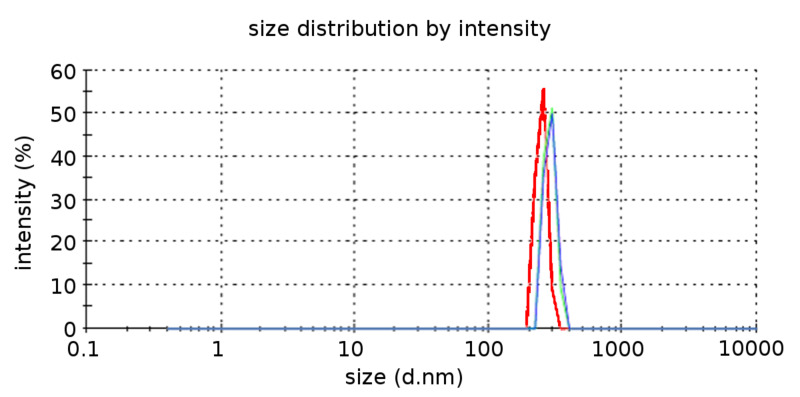
Size distribution measurements of a 0.08 M NaCl solution after sonication.

This raises the question raised as to whether the nanoparticles were formed in the solution during sonication and then deposited on the surface of the substrate, or whether they crystallized directly on the surface. To confirm the presence of the nanoparticles in the solution and estimate their size, DLS measurements of particles in the solution after the sonication process were performed. The results are summarized in the Figure 8. It was found (data not shown) that the size of the ionic NPs was dependent on the concentration of the ionic material. The use of a higher initial concentration of inorganic salts (>0.5 M) resulted in the appearance of larger particles (400–500 nm). The DLS of the original was measured and no particles were detected.

**Figure 9:**
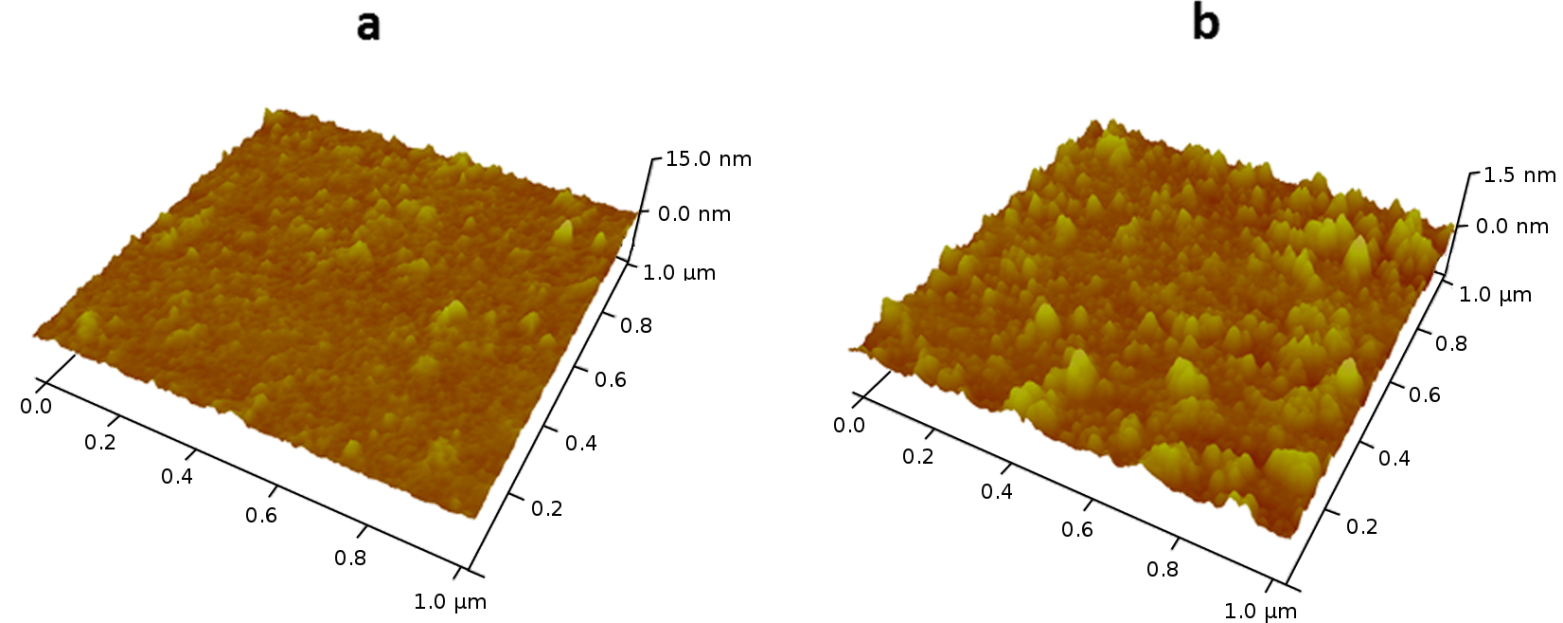
3D AFM image of the microscope slide after sonication (30 min) in doubly distilled water; (b) 3D AFM image of the microscope slide coated with CuSO_4_ NPs (0.125 M CuSO_4_, 30 min).

The surface morphology of deposed nanoparticles was also characterized by the AFM method. The 3D AFM images of the standard glass slide before and after sonication with CuSO_4_ are presented in Figure 9. The size of the obtained nanoparticles ranges from 15 nm to 75 nm. The mean square roughness of the glass with sonochemically deposited nanoparticles is higher (1.40 nm) than the roughness of the bare microscope glass slide (0.652 nm). These findings give further proof of the ability of the sonochemical method to deposit inorganic nanoparticles from aqueous solution on the microscope glass slides and parylene-coated glass by a one-step, ultrasound-assisted procedure. A comparison of the SEM and AFM results measured for the same sample, as illustrated in Figure 7b and Figure 9b, reveals very similar particles sizes (70 nm).

### Leaching studies

**Figure 10:**
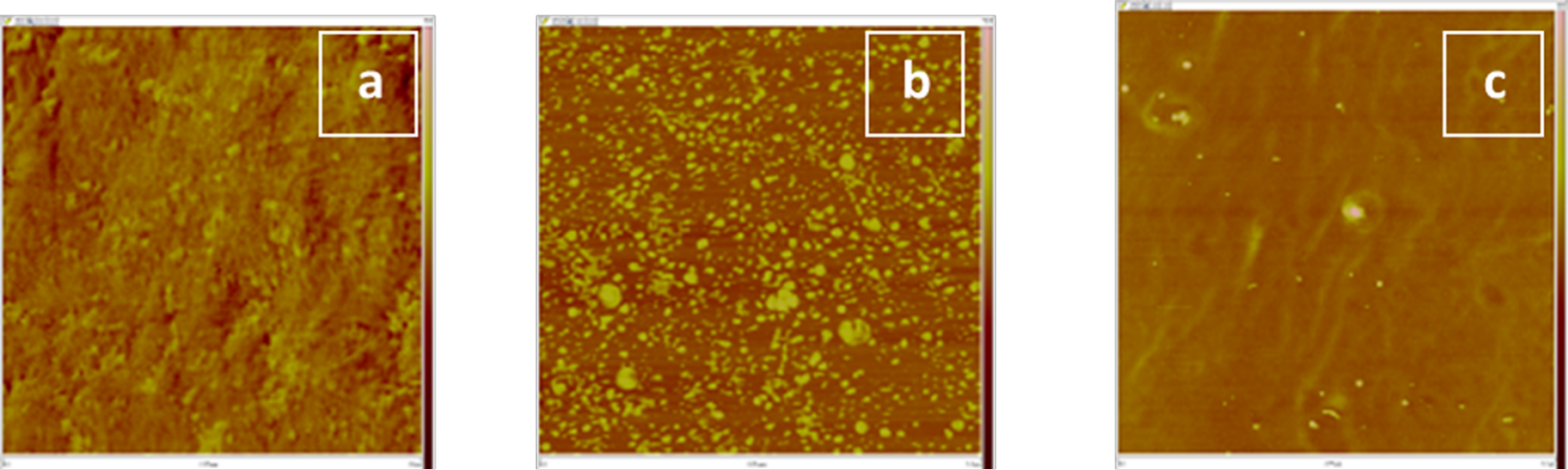
The AFM images of (a) parylene-coated glass slide before the sonication process; (b) parylene-coated glass slide after sonication in the presence of NaCl; (c) the same glass slide after 24 h of the leaching process.

To examine the strength of anchoring of the NPs to the substrate, some control experiments on the release of the ionic compounds into the surrounding environment were conducted. Namely, we placed the coated glass slides in the doubly distilled water at room temperature for different periods of time (from 2 to 96 h) under continuous stirring. The methods we used for revealing the leaching of nanoparticles were ICP and AFM. The ICP measurements showed that after 24 h the concentrations of Na^+^ and Cl^–^ in water were 0.04 and 0.08 mg/L, respectively, and after 48 h the concentrations of Na^+^ and Cl^–^ increased to 9.3 ± 0.4 and 9.1 ± 0.3 mg/L, respectively. There is almost no change in the ion concentrations in water between 48 and 96 h. The surface morphologies of the coated glass slides after the leaching experiments, obtained with the AFM method, are shown in Figure 10. The obtained results indicated that after 24 h of leaching the nanoparticles were still present on the substrates. The complete leaching of nanoparticles occurred after 96 h. The same trend was observed for glass slides coated with CuSO_4_ and KI nanoparticles.

### Mechanism of the sonochemical deposition

The sonochemical mechanism by which the nanoparticles were deposited on the substrates was discussed previously and related to the creation of microjets and shock waves as the after effects of acoustic cavitation [11–12].

In the previous studies performed by our group, the deposition of a large variety of nanoparticles on different types of substrates was achieved by using the sonochemical method. In general, there are two main routes for the deposition:

(1) The coating of substrates with nanoparticles that were directly synthesized during the sonochemical reaction (in situ mode of deposition) [17–18].

(2) The coating of substrates with preliminarily synthesized nanoparticles or those purchased from commercial sources ("throwing stones" mode of deposition) [19].

The present work describes an additional route for the deposition of nanoparticles. It was found that the sonication of aqueous solutions of inorganic compounds (NaCl, CuSO_4_, KI) resulted in the formation of nanoparticles of the examined salt. In addition, it was found that if this process occurred in the present of glass slide, the nanoparticles formed are deposited onto it.

**Figure 11:**
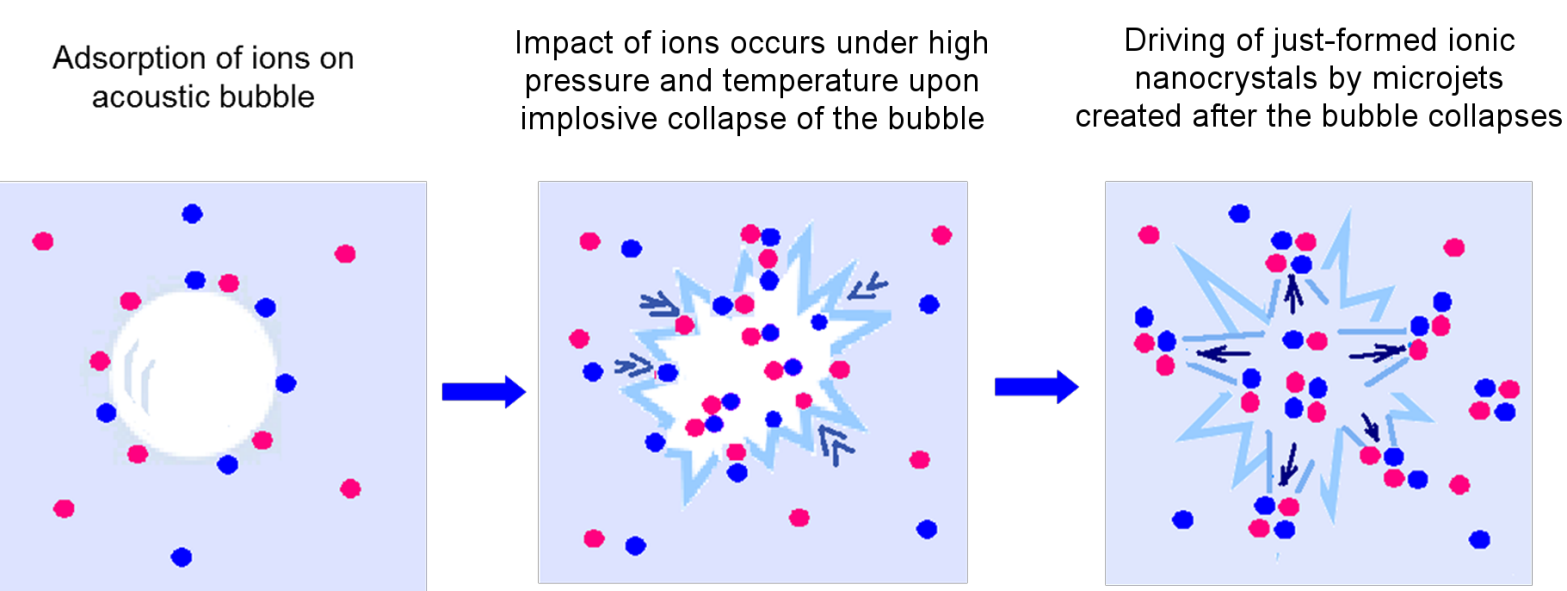
Scheme of the sonochemical deposition of inorganic salt nanoparticles on the solid substrate.

The proposed mechanism for the described process is the following: When ultrasonic treatment is applied to the aqueous solution of an inorganic salt, the ions of the salt are absorbed on or near the formed acoustic bubbles [20]. When the cavity collapse occurs, the absorbed ions are exposed to extreme, localized conditions of temperature and pressure. As a result, these ions impact one another and nanoparticles of the inorganic salt are produced as a result of the influence of localized higher temperatures and pressures. According to Kordylla et al. [21] the nucleation work needed for crystallization is strongly reduced by the presence of a liquid–gas interface (the bubble surface), therefore the presence of the bubble surface accelerates the crystal nucleation. It is worth mentioning that Lepoint-Mullie and co-workers detected visible emission spectra in the vicinity of resonance lines of alkali metals from acoustically cavitating aqueous and 1-octanol solutions of NaCl and RbCl [22]. They showed that the emission from the alkali metals arose from the gas phase of the bubbles. The ions reached the hot center of the bubble by the flow of the liquid towards the center upon their collapse. In our case, cavitation occurs not only in pure liquid, but also in liquid near a solid surface (glass slides) and it was found that the presence of a solid surface increases the nucleation rate [23]. The generated high-speed jets of the liquid, formed after the collapse of the bubble, throw the formed nanoparticles at high speed toward the glass slides. The proposed mechanism for the sonochemical deposition of inorganic nanoparticles is presented schematically in Figure 11.

## Conclusion

The NaCl, CuSO_4_ and KI nanoparticles were deposited on the surface of bare or parylene-coated glass slides and silicon wafer or parylene-coated silicon wafer slides by means of ultrasonic (sonochemical) treatment. The size of the observed nanoparticles and their distribution on the substrate were affected by the concentration of the mother solution and the time of exposure to the sonication. The most homogeneous coating was obtained when the initial concentration of inorganic salts was in the range 0.05–0.125 M and the time of the reaction was 30 min. In addition, it was also observed that after 24 h of leaching the nanoparticles are still present on the slides, and complete leaching of the nanoparticles occurred only after 96 h. The obtained results clearly demonstrated the efficiency of the ultrasound-assisted method for the deposition of inorganic salt nanoparticles on different types of solid surfaces. This technique can be used to introduce improved properties in terms of electroactivity and conductivity to bare and parylene coated glass slides through such surface modifications.
